# Ironing out differences in attenuation and blooming artifact in acute stroke thrombi

**DOI:** 10.1038/s41598-024-83916-0

**Published:** 2025-01-02

**Authors:** Aglae Velasco Gonzalez, Dennis Görlich, Boris Buerke, Cristina Sauerland, Norbert Meier, Manfred Fobker, Ray McCarthy, Astrid Jeibmann, Walter Heindel, Andreas Faldum, Harald Kugel

**Affiliations:** 1 Clinic for Radiology, Neuroradiology, University of Münster and University Hospital Münster, 48149 Münster, Albert-Schweitzer-Campus 1, Building A1 Germany; 2 Institute of Biostatistics and Clinical Research, University of Münster, 48149 Münster, Schmeddingstraße 56 Germany; 3 Center for Laboratory Medicine, University Hospital Münster, 48149 Münster, Albert-Schweitzer-Campus 1, Building A1 Germany; 4 Institute of Neuropathology, University Hospital Münster, 48149 Münster, Pottkamp 2 Germany; 5 Medical Physics, Clinic for Radiology, University of Münster and University Hospital of Münster, 48149 Münster, Albert-Schweitzer-Campus 1, Building A1 Germany; 6 Cerenovus, Galway Neuro Technology Centre, Galway, Mervue Business Park Ireland

**Keywords:** Blood clot, Helical CT, Red blood cells, Decision trees, Iron, Stroke, Magnetic resonance imaging, Cardiovascular diseases

## Abstract

This study aims to improve our understanding of acute ischemic stroke clot imaging by integrating CT attenuation information with MRI susceptibility signal of thrombi. For this proof-of-principle experimental study, fifty-seven clot analogs were produced using ovine venous blood with a broad histological spectrum. Each clot analog was analyzed to determine its RBC content and chemical composition, including water, Fe III, sodium, pH, and pO2. Non-contrast CT and a susceptibility-weighted MRI sequence were used for imaging. The study found that RBC content correlated more accurately than iron content with clot attenuation on CT. There was a strong correlation between Fe III content and RBC percentage in clots. Specifically, changes in RBC content accounted for 64% of the variance in Fe III content (R2 = 0.640; *p* < .0001). Thrombi with blooming artifacts (BA) displayed higher attenuation on non-contrast CT than those without (73.4 vs. 40 HU, *p* < .0001) and had the highest RBC and iron contents. The cut-off value of 1242 µg/g of iron predicted blooming artifacts with high sensitivity and specificity. The pH level strongly affected the appearance of blooming artifacts, particularly for negative clots with high RBC content. These findings provide significant insights into the imaging behavior of acute ischemic stroke clots across both imaging modalities and could potentially improve the diagnosis and treatment of acute stroke patients. Furthermore, these results open the possibility for future research aimed at developing pH-modulated therapeutic strategies based on the acid-base state of thrombi.

**Supplementary Information:**

The online version contains supplementary material available at 10.1038/s41598-024-83916-0.

## Introduction

Patients in the acute stroke decision pipeline are typically evaluated using only one imaging modality, either CT or MRI. For that reason, the lack of correlation between CT and MR imaging features over time has made it challenging to understand the variable imaging behaviors of acute ischemic stroke clots.

Several studies have attempted to understand the imaging features of acute ischemic stroke clots based on their red blood cell (RBC) content^1–3^. The paramagnetic susceptibility signal is an important clot feature on susceptibility-weighted images (SWI) or T2*-weighted gradient echo sequences in MR imaging (3–5). The magnetic susceptibility of matter modifies an external magnetic field, slightly increasing it if the susceptibility is positive, i.e., paramagnetic, or decreasing it if the susceptibility is negative, i.e., diamagnetic. If a thrombus in a blood vessel is more paramagnetic than the surrounding tissue, an inhomogeneity arises in the magnetic field over the vessel, resulting in rapid dephasing of the transverse component of the spins after an MR excitation pulse, i.e., in a very short T2* time, which leads to signal loss in strongly T2*-weighted imaging sequences. When there is a large difference in the susceptibility of the clot and that of the surrounding tissue, field inhomogeneity can cause signal loss that can exceed vessel volume. In acute stroke patients, this effect is commonly known as a blooming artifact (BA) or susceptibility vessel signal.

In daily practice the susceptibility signal is generally evaluated qualitatively (4). However, it is possible to measure specific T2* relaxation times for different histological parts of thrombi^4,5^. Thrombi have exhibited a wide range of possible susceptibility values, leading to T2*-values from 16 to 268 ms^4^. This range of T2* times is broader than what would be expected from changes in blood oxygenation alone, as well-oxygenated blood has a T2* of 200 msec and deoxygenated blood 100 msec^6^.

The proportion of acute ischemic stroke clots displaying BA ranges from 30 to 83%^7–10^. They are often associated with a high RBC content, although the average RBC content does not usually exceed 40%^3,11,12^, and some studies have found no association^13^. Along these same lines, the degree of attenuation on non-contrast CT (NCCT) of clots is closely related to the RBC content, but hyperattenuated clots without high RBC content have also been reported^1,3^.

These disparities in previous studies show that there is still a need to more fully understand how acute clot susceptibility varies with histological and chemical composition. The objective of this study is to expand our knowledge of clot imaging in acute ischemic stroke by integrating the CT attenuation and MRI susceptibility information for clots with their histological and chemical characteristics.

## Results

### General results

A total of 57 clot analogs with an RBC content of 71.4% (IQR: 26, 95; range: 0, 99) and a fibrin content of 28.6% (IQR: 4.8, 74; range: 0, 100) were studied by CT and MRI. The median iron content was 1102 µg/g (IQR: 412, 1625; range: 9, 3005), and the water content was 69.8% (IQR: 42.1, 84; range: 14.3–88.1). Thrombus pH ranged from 6.449 to 7.917, with a median of 7.204 (IQR, 6.924, 7.480; range 6.449–7.917, 2 missing data points). All clots were homogeneously well oxygenated, with a median pO2 of 205 mmHg (IQR 196, 239; range 178–264).

**Fig. 1:**
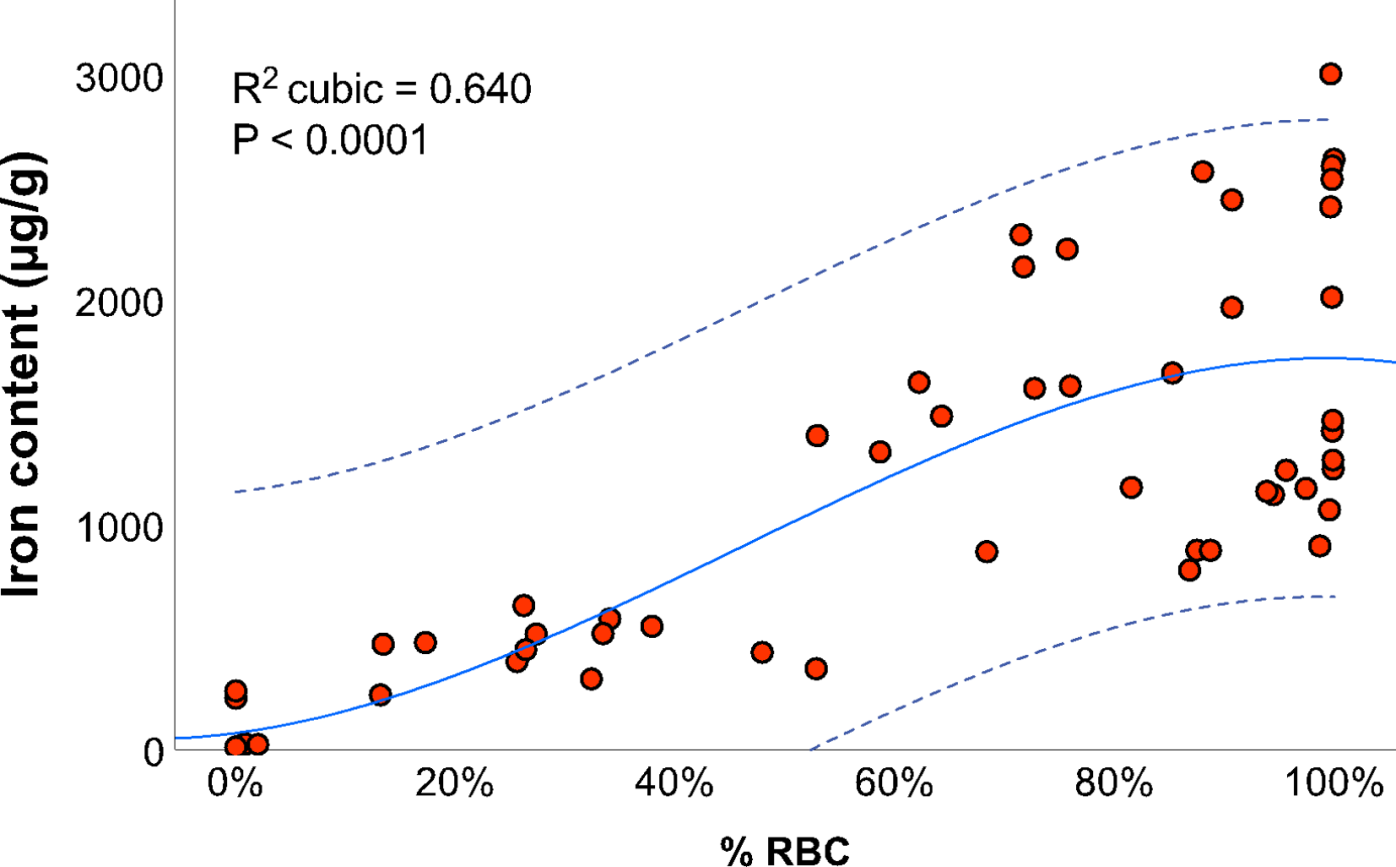
Distribution of RBC and iron contents in the clot analogs. The Fe III content was calculated based on the cubic regression model as follows: Iron = 74.36 + (6.4 x RBC^2^) + (0.38 x RBC^2^) – (2.76^(−3)^x RBC^3^). Broken lines indicate the 95% CI of the cubic prediction (continuous blue line). RBC: red blood cells.

There was good correlation between the Fe III content and the percentage RBC in the clots (Spearman ρ = 0.804, *p* < .0001). Changes in the RBC content accounted for 64% of the variance in the Fe III content among the thrombi (R^2^ = 0.640; *p* < .0001). Figure 1 depicts the wide range of iron concentrations for the clots, especially in those consisting of more than 60% erythrocytes. There was a strong positive correlation between CT clot attenuation (increased HU) and percentage RBC, with a correlation coefficient (Spearman ρ) of 0.891 (CI 95%, 0.818–0.935). The correlation coefficient for the iron content was slightly lower (0.790, CI 95%, 0.662–0.873).

**Fig. 2:**
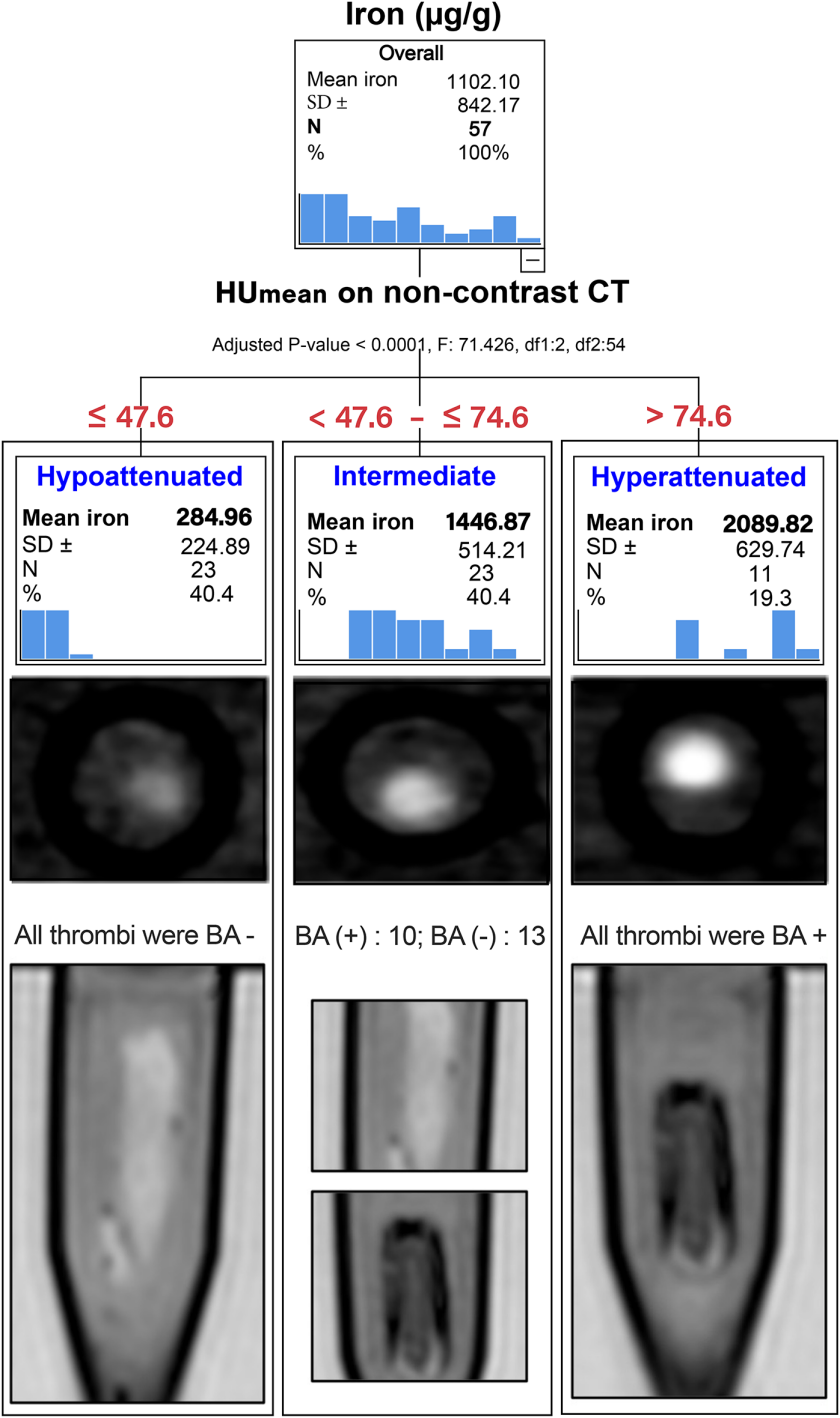
Decision tree for predicting iron content from non-contrast CT clot attenuation and correlation with the SWI signal. Clot iron content could be divided into three groups based on clot attenuation values on non-contrast CT. Below, clot group correlation with the presence or absence of blooming artifact (BA) on MR imaging of each CT group. HU: Hounsfield Units (in red).

We developed a decision tree to determine which HU measurements classified the iron content most accurately. The decision tree identified three groups of clots based on the mean axial HU value (*p* < .0001). All hyperattenuated thrombi (HU > 75, *n* = 11) displayed the BA and had a mean iron content of 2090 ± 630 µg/g, while those with hypoattenuation (HU ≤ 47.6, *n* = 23) did not show any blooming artifact (BA negative) and had a lower iron content (285 ± 224 µg/g). The intermediate group, ranging from 49 to 75 HU (*n* = 23), included 10 BA negative and 13 BA positive thrombi with an average iron content of 1147 ± 514 µg/g. Table 1 presents the compositional analysis for each group, and the decision tree is displayed in Fig. 2. For visual representation of the variable distributions in Table 1, boxplots are available in the supplemental materials (Figure S1).

**Table 1:** Composition characteristics of clot groups for predicting iron content based on attenuation. For visual representations of data distributions, please refer to the boxplots provided in the supplemental materials (figure S1).

Parameter	Hypoattenuated (*n* = 23)	Intermediate (*n* = 23)	Hyperattenuated (*n* = 11)	*P*-Value
Attenuation cut-offs (HU)^1^	≤ 47.6	47.6 – ≤ 74.6	> 74.6	< 0.0001
Attenuation (HU)				
Median IQR Range	37.2 32.7, 40.5 29–47.6	62.7 55.5, 70.7 52–74.6	82.5 80, 85.1 75.1–90.7	< 0.0001
RBC				
Median IQR Range	13.4 0.04, 32.4 0–53	85.2 71.4, 94.4 53–99.9	99.7 95.6, 99.8 88–99.8	< 0.0001
Iron (µg/g)				
Median IQR Range	315 18, 476 9–641	1324 1067, 1675 799–2625	2414 1417, 2570 1242–3005	< 0.0001
Water				
Median IQR Range	84.7 65.2, 87 14.3–88	69.8 40.6, 74 25–80.7	63 45.3, 65.3 39–69	0.0010
pH ^2^				
Median IQR Range	7.408 7, 7.556 6.459–7.917	7.177 6.738, 7.370 6.519–7.600	7.204 6.956, 7.350 6.932–7.450	0.6134
Na (mmol/L)				
Median IQR Range	22.3 17.8, 32.2 15–154.7	17.1 14.2, 151.4 10.7–155	20.3 14.5, 31 11.1–153.1	0.3709
pO2 (mmHg)				
Median IQR Range	197 195, 236 178–256	202 197, 231 191–254	225 205, 262 194–264	0.0598

All parameter values are percentage values unless otherwise specified ^1^Mean clot attenuation from 1 mm-axial non-contrast CT reconstruction. HU: Hounsfield Units; RBC: red blood cells. ^2^There were two missing datas in the group of hypoattenuated clots. *P-Value: Mann-Whitney U Test.

## Susceptibility signal

In total, 24 thrombi exhibited BA and 33 did not (compositional analysis in Table 2). Thrombi with BA displayed higher attenuation (median HU: 73.4, IQR: 36.2, 55.3) than those without (median HU: 40, IQR: 62, 82) (*p* < .0001) as well as a high RBC content (median of 95% vs. 27.3%, *p* < .0001; mean of 87% ± 16% vs. 38.1% ± 34%) and iron content (median: 1626 vs. 469 µg/g, *p* < .0001). There were no differences in water content between the two groups. A detailed visualization of the distribution of these variables can be found in the boxplots provided in the supplemental materials (Figure S2).

**Table 2:** Overall characteristics of thrombi with and without blooming artifact on MRI. Boxplots illustrating the distribution of these variables are available in the supplemental materials (figure S2).

Parameter	BA-negative (*n* = 33)	BA-positive (*n* = 24)	*P*-Value
Attenuation (HU)^1^			
Median IQR Range	40 36.2, 55.3 29–72.5	73.4 62, 82 52–90.7	< 0.0001
RBC			
Median IQR Range	27.3 0.9, 71.4 0–99.5	95 74.2, 99.7 52.9–99.9	< 0.0001
Iron (µg/g)			
Median IQR Range	469 230, 881 9–2290	1626 1361, 2430 906–3005	< 0.0001
Water			
Median IQR Range	75.4 41, 86 14.3–88.1	66.4 55.2, 72.7 35.3–80.7	0.117
pH			
Median IQR Range Missing	7.408 7.083, 7.580 6.45–7.917 2	7.067 6.781, 7.243 6.519–7.45 -	0.0051
Na (mmol/L)			
Median IQR Range	20.4 17, 27 12.9–155	20.2 13.8, 159 10.7–154.3	0.3241
pO2 (mmHg)			
Median IQR Range	197 195, 235 178–256	219 200, 252 191–264	0.024

All parameter values are percentage values unless otherwise specified. ^1^Mean clot attenuation from 1 mm-axial non-contrast CT reconstructions. HU: Hounsfield Units; RBC: red blood cells; BA: blooming artifact. P-Value: Mann-Whitney U Test.

The threshold value of iron content resulting in the appearance of BA was 1242 µg/g (*p* < .0001). This finding had 93.9% sensitivity and 91.7% specificity as determined by Optimal Binning using MDLP. Figure S3 in the supplementary materials depicts the decision tree used for iron threshold selection.

Clots with intermediate attenuation (47.6 – ≤ 74.6 HU, *n* = 23) had similar CT attenuation and histological characteristics regardless of their BA status. Both BA-negative (*n* = 10) and BA-positive (*n* = 11) were RBC-rich thrombi (88% vs. 75.6% RBC, respectively, *p* = .3128) and displayed similar CT attenuation (62.6 vs. 62.7 HU, respectively, *p* = .9273). However, BA-negative clots with intermediate CT attenuation contained appreciably less iron (median 1108 vs. 1626 µg/g, *p* = .0255) and water (median 40.7% vs. 72.7%, *p* = .0255) and had a higher pH (median 7.440 vs. 6.824, *p* < .001) than the BA-positive thrombi with intermediate CT attenuation. Detailed results can be found in Table S1 in the Supplementary Materials.

## Characterization of two different BA-negative clots

As explained above, two groups of clots that did not display the BA and represented two histological extremes were identified. The first group (*n* = 23) consisted of clots with a hypodense appearance on NCCT (belonging to the hypoattenuated CT group, 37.2 HU) with a low RBC content (median 13.4%). The second group (*n* = 10) consisted of the BA-negative thrombi from the intermediate CT group, with a notably elevated percentage RBC (median 88%) and higher attenuation values (62.6 HU). The composition of these two histological types of BA-negative clots is presented in detail in Table 3 and further illustrated with boxplots in the supplemental materials (Figure S4).

**Table 3:** Two histological types of blooming artifact-negative thrombi. Boxplots iustrating the distribution of these variables are available in the supplemental materials (figure S4).

Parameter	BA–negative thrombi		
	Intermediate attenuation^1^ (*n* = 10)	Hypoattenuated^2^ (*n* = 23)	*P*-Value
Attenuation (HU)^1^			
Median IQR Range	62.6 55.9, 70 53.5–72.5	37.2 32.7, 40.5 28.9–47.6	< 0.0001
RBC (%)			
Median IQR Range	88 81.5, 93.8 68.3–99.5	13.4 0.04, 32.4 0–53	< 0.0001
Iron (µg/g)			
Median IQR Range	1108 887, 1166 799–2290	315 18, 476 9–641	< 0.0001
Water (%)			
Median IQR Range	40.7 38.7, 71 25–73.8	84.7 65.2, 86.8 14.3–88	0.002
pH			
Median IQR Range	7.440 7.263, 7.580 7.124–7.6	7.408 7.000, 7.556 6.449–7.917	0.348
Na (mmol/L)			
Median IQR Range	17.9 16.8, 23 12.9–155	22.3 17.8, 32.2 15–154.3	0.207
pO2 (mmHg)			
Median IQR Range	199 195, 205 193–250	197 195, 236 178–256	0.743

^1^Intermediate CT-attenuation group BA-negative thrombi (10 of 23). ^2 ^Low CT attenuation group BA-negative thrombi (*n* = 23). All hypoattenuated clots were BA negative. The data for this group already presented in Table 1 have been repeated here for comparison. HU: Hounsfield Units; RBC: red-blood-cells. BA: blooming artifact. P-Value: Mann-Whitney U Test.

The analysis revealed a distinct common factor between the two types of BA-negative clots: their pH level. Interestingly, the pH was more alkaline in the BA-negative thrombi than in the BA-positive thrombi, and this difference was even more pronounced for the group of clots with intermediate attenuation (7.440 vs. 6.824, respectively, *p* < .001). Furthermore, pH was shown to be the most significant factor in determining the presence or absence of BA in the group of clots with attenuation values between 47.6 – ≤ 74.6 HU (*n* = 23), with a regression coefficient of −10.454 (95% CI 0–0.592, *p* = .039). The model did not take the percentage RBC and iron content or the water content into account.

Finally, we conducted an ROC analysis to investigate the variables that influenced the presence or absence of BA for the 57 thrombi. In line with previous analyses, the results showed that iron content was a better predictor of the paramagnetic susceptibility signal than RBC content, as evidenced by the areas under the curve of 0.949 and 0.875, respectively. Furthermore, we also performed an ROC subgroup analysis of RBC-rich clots with attenuations between 47.6 – ≤ 74.6 HU (intermediate attenuation group, *n* = 23). Interestingly, the RBC content in this subgroup lost and water content gained predictive power, with an AUC value of 0.777 (*p* = .0049). Moreover, pH values had low predictive power for BA-positive clots (AUC: 0.069). Conversely, the pH value exhibited high predictive capacity for the opposite case scenario, the BA-negative thrombi (AUC: 0.931 ± 0.051, 95% CI (0.830, 1.000), *p* = .0005). Thus, the higher the pH value, the more probable a BA-negative thrombus. The results of the ROC analysis are presented in Fig. 2, and the AUC values are given in Table 4.

**Table 4:** Area under the curve for blooming artifact-positive thrombi.

All thrombi (*n* = 57)				
Parameter	Area	Std. Error	CI	*P*-Value
Iron (µg/g)	0.949	0.031	0.889–1.009	< 0.0001
% RBC	0.875	0.045	0.787–0.963	< 0.0001
% Water	0.402	0.080	0.245–0.559	0.2202
pH	0.278	0.070	0.140–0.416	0.0016
pO2 (mmHg)	0.655	0.075	0.508–0.802	0.0386
Intermediate attenuation group thrombi (*n* = 23)				
Iron (µg/g)	0.777	0.111	0.559–0.995	0.0128
% RBC	0.369	0.122	0.131–0.607	0.2818
% Water	0.777	0.098	0.584–0.970	0.0049
pH	0.069	0.051	−0.032–0.170	0.0016
pO2 (mmHg)	0.658	0.118	0.427–0.889	0.1808

P-Value under the non-parametric assumption. ROC: Receiver operating characteristic. HU: Hounsfield Units; RBC: red blood cells; CI: asymptotic 95% confidence interval.

## Discussion

### Causes of blooming artifact in clots

Iron was the primary factor determining BA. Thrombi displayed a BA when the Fe III content was above 1242 µg/g (93.9% sensitivity and 81.7% specificity). One significant source of ferric iron (Fe III) in blood is methemoglobin, the oxidized form of deoxyhemoglobin that cannot bind oxygen^14^, responsible for susceptibility artifacts in acute hemorrhage^15^. The rate of methemoglobin formation in clots under normal arterial flow is unknown, but studies of clot analogs indicate a very low rate of production, even lower in well-oxygenated conditions^5^. Fe III can be found in 50–70% of oxyhemoglobin in blood^16^and in small amounts in plasma transferrin^17^. Due to the high levels of oxygenation in our clot samples, it is challenging to determine the origin of the Fe III in our study. While significant oxidative processes are unlikely to occur in these circumstances, other environmental factors may affect the atomic state of the iron, as discussed below based on some of our results.

The second most important factor in predicting BA in our study, after the iron content, was the RBC content. Previous studies have shown that high hematocrit levels and high RBC contents reduce T2 and T2* times. However, blooming occurred when both variables exceeded a certain threshold. The threshold for hematocrit was 40%, and the threshold for the RBC content was 54% ^4,5^, which is consistent with our results. We found that the RBC composition of BA-positive thrombi could range from 50% to over 90% ^11^. Unfortunately, this range may include acute ischemic stroke thrombi with varying mechanical properties^18^, so the BA-artifact is of only limited use in selecting material for mechanical thrombectomy.

While this study highlights the roles of RBC and iron content in imaging characteristics, other factors, including cell packing density, iron distribution, and interactions with the thrombus environment, may also significantly impact clot susceptibility and require further investigation.

### RBC-rich thrombi without a BA

The iron content in the thrombi containing more than 60% RBC was highly variable in our study. This finding may explain why some RBC-rich thrombi do not exhibit BA on MRI. Clinically, this phenomenon could occur in acute stroke patients with anemia, a common condition among elderly individuals^19^, potentially leading to RBC-rich thrombi with a negative BA on MRI. However, clinical validation is still required to confirm this hypothesis.

Additionally, we identified two groups of clots without a BA: one with a low percentage RBC (13%), very low iron content (315 µg/g), and hypoattenuation on non-contrast CT (37 HU), and another RBC-rich group (88% RBC) with high iron content (median 1108 µg/g), and high attenuation (62 HU). Both types of BA-negative clots exhibited a slightly more alkaline pH (7.4) than the BA-positive clots (pH 6.8, *p* < .001). Moreover, pH values had a high predictive capacity for BA-negative thrombi (AUC: 0.931) in the range of 47.6 – ≤ 74.6 HU.

Blood pH ranged from 7.35 to 7.45 and reflected the hydrogen ions concentration^20^. Hemoglobin’s capacity to bind oxygen increases as the pH rises^21^. Therefore, oxyhemoglobin (more diamagnetic) is prevalent in basic pH environments^22^, as could be expected for the BA-negative clots in our study. In addition, pH levels affect the magnetic behavior of methemoglobin. Low-spin methemoglobin is formed at a basic pH of 7.4 and has less paramagnetic susceptibility^23,24^. Therefore, both these factors, mediated by a more alkaline pH environment, may have a synergistic effect in decreasing the expected paramagnetic susceptibility of RBC-rich clots. These findings suggest that pH influences thrombi under experimental conditions, but in vivo thrombi in contact with blood at physiological pH may behave differently, warranting further investigation.

### pH of BA-positive clots

Our study discovered that thrombi positive for BA were typically more acidic, falling within a pH range of 6.5–7.3, outside the normal pH range of blood. This acidic pH can have a significant impact on the hemoglobin molecule. It can alter the hemoglobin’s interaction with the medium, induce structural changes^25^, affect water exchange across the RBC membrane^26,27^, and ultimately result in a loss of oxygen transport function as the hemoglobin dissociates into two dimers when the pH lies outside the normal range^21,28^. These changes can significantly increase the magnetic susceptibility of the blood, leading to B_0_ microinhomogeneities that can strongly influence T2* relaxation times.

### Limitations

Firstly, the clots used in our study were not as complex as in vivo clots. We focused on well-oxygenated thrombi, assuming minimal effects from varying hemoglobin oxidation states. This allowed us to investigate the significance of other factors such as iron, red blood cells, and pH levels. However, the paramagnetic properties of clots are influenced by multiple factors, including compositional changes under flow conditions or aging time, which were not explored here and merit further exploration in future studies. While the clots were imaged consistently within 20 h of preparation and maintained at 3 °C throughout, potential changes in hemoglobin oxidation state during this period cannot be entirely excluded. However, formation of methemoglobin in experimental thrombi is minimal in this timeframe (0.042%/hour during the initial 30 h)^29^, thus, the imaging findings in this study are primarily reflective of the clot’s original composition rather than degradation products. The results of the histological and laboratory analyses are representative of the state of the clots at the time of imaging. In our study, we used calcium chloride to restore calcium levels after chelation by sodium citrate, facilitating clot formation. While this addition could theoretically affect radiographic density, a significant effect is unlikely due to the expulsion of calcium into the serum and our consistent application across all clots. However, we did not directly analyze calcium content, so further investigation is needed to clarify this aspect. Further research is needed to fully understand the role of pH in thrombus formation and susceptibility in vivo. It should be noted that while we hypothesize that pH may influence the atomic state of iron, this was not directly assessed in our study and warrants further investigation. Additionally, the distribution of iron atoms within the clot may depend on conditions during clot formation. Furthermore, variations in red blood cell size across mammalian species may have caused slight shifts in the study’s results, as human blood shows higher Mean Corpuscular Volume values. While clinical laboratory results tend to be precise, the minimum sample size for reliable analytical results remains unclear. As this study focused in clot analogs, caution is warranted when interpreting these findings in a clinical context, and further validation in vivo is essential.

## Conclusions

Clot imaging is important when selecting appropriate materials for mechanical thrombectomy, as clot composition can impact successful outcomes. This experimental study with clot analogs demonstrates that the presence or absence of the susceptibility signal on MR imaging of clots primarily depends on the iron content rather than on the RBC content. Additionally, we were able to identify two types of clots without blooming artifacts, namely “typical” clots with low RBC and iron contents and RBC-rich clots. Interestingly, clot pH was a determining factor for predicting a lack of paramagnetic (or, more precisely, less diamagnetic) susceptibility of RBC-rich clot analogs. Finally, attenuation of thrombi on CT was not a reliable predictor of the susceptibility signal on MRI, except where clots were either extremely hypoattenuated (fibrin-rich) or extremely hyperattenuated (more than 90% RBC). These findings are based on experimental clot analogs and may not fully reflect the complexities of in vivo conditions, warranting further investigation.

## Methods

Our objective in this experimental proof-of-principle study was to investigate the relationship between CT attenuation and the MRI susceptibility signal of human-like acute ischemic stroke thrombi. A previous study of clot attenuation on CT served as a basis for the current analysis^1^, and results on other standard stroke MRI sequences have been published elsewhere^30^. The current analysis does not overlap with any relevant published MRI features. This study was approved by the University of Münster, Germany. Clot extraction from ovine species was performed in accordance with relevant guidelines and regulations. Methods are reported in accordance with ARRIVE (Animal Research: Reporting of In Vivo Experiments) guidelines.

### Study design

We used 57 clot analogs with differing compositions produced from ovine venous blood^31^. Each clot analog underwent neuropathological analysis for RBC percentage and laboratory tests for water and iron (III) contents, sodium, pO2, and pH values. The clots were prepared and positioned in a CT and MRI compatible phantom and imaged by a 0.4 mm axial non-contrast scan with CT (Somatom Definition Flash) and using a susceptibility-weighted imaging sequence with MRI (Philips Ingenia 1.5T MRI). The time elapsing between clot production and CT imaging was 12 h and between CT and MRI imaging was 6 h. We evaluated CT clot attenuation, laboratory results, and the presence of a blooming artifact (yes/no). Two of the authors (HK, senior medical MR physicist, and AVG, senior neuroradiologist) were in charge of the MRI clot measurements, and two other authors (AJ, senior neuropathologist, and AVG, senior neuroradiologist) carried out the histological analysis of each clot. In both cases, a consensus was reached regarding the final value or classification. The data assessment was conducted in a blinded manner to avoid any influence by radiological or histological information using independent databases that converged at the point of statistical analysis.

### Clot production

Venous blood was obtained from the jugular vein of ovine species at a licensed facility (Ash Stream Ltd., Mayo, Ireland) with trained veterinarians to avoid animal suffering. The thrombi were produced by Cerenovus (Mechanical and Industrial Engineering Department, Galway-Mayo Institute of Technology, Galway, Ireland) with different RBC fractions from blood mixtures with hematocrit levels of 84%, 20%, 5%, 1%, and 0% using methods previously described in detail and published elsewhere^1,30–32^. The thrombi were transported and stored in their own serum in sealed containers at approximately 3 °C until use.

### Histological analysis

Histological evaluation of each of the clots imaged was based on hematoxylin-eosin and Martius Scarlet Blue stains. Clot cores were photographed under x40 magnification (Olympus BX43 microscope + digital camera). ImageJ software (version 1.52a) was used for quantitative threshold-based automatic measurement of the percentage RBC.

### Clinical laboratory analysis

The water content was determined by drying the samples at 90 °C using the standard protocol. The iron (III) was measured by spectrometry. To determine the water content, samples were weighed (ME235P, Sartorius, Göttingen, Germany) before and after drying at 90 °C for three days. For the iron determination samples were dried at 70 °C overnight to reach a constant mass and transferred to a 500-µl PFA vessel (AHF-Analysetechnik AG, Tübingen, Germany). An amount of 100 µl of HNO_3_ (60% v/v) was added and incubated overnight at room temperature. Before measurement, samples were incubated at 70 °C for 90 min, allowed to cool, and diluted 10-fold with H_2_O. Next, 10 µl of the solution was placed directly in a graphite furnace for atomic absorption spectrometry (AA6300, Shimadzu, Kyoto, Japan). For each determination, measurements were carried out in triplicate. The recommended operating conditions for the spectrometer were: hollow-cathode lamp current: 12 mA; absorption wavelength: 248.3 nm; BGC-D2-Modus; gap width: 0.2 nm. Working solutions containing 0, 5, 10, 15, and 20 µg/l FeCl_3_ were used as standard solutions. The Limit of Quantitation, Limit of Detection, and the linear range for Fe analysis were 0.5 µg/l, 0.1 µg/l, and 0.5–20 µg/l, respectively. The intra and inter-day precision of the assay, expressed as a coefficient of variation, ranged from 2.8 to 6.5%. For determination of pH, pO_2_ and sodium 10 mg clot tissue and a 1-cm dry ice (3 mm pellets) layer was placed in a mill grinding chamber (TUBE MILL CONTROL, IKA Werke, Staufen, Germany). After homogenization (2 min) the sample was transferred into an Eppendorf tube and 70 µl distilled water added after evaporation of ice, followed by centrifugation at 13,000 rpm. The supernatant was analyzed using a commercial blood gas analyzer (ABL800 FLEX, Radiometer, Copenhagen, Denmark).

### Specimen preparation and imaging protocol

Specimen preparation and the CT imaging protocol have been published elsewhere^1^. To summarize, before imaging, clot analogs were placed parallel to the long axis of an Eppendorf tube and fixed centrally in 2% agarose, carefully avoiding air bubbles at the interface. Each Eppendorf tube was placed in a plexiglass holder and immersed in a plastic container filled with 2.5 L water + 0.2 mL Gadovist^®^ (gadobutrol, 1.0 mmol/mL) contrast agent. This phantom was CT and MRI-compatible. Clots were first imaged by NCCT using the following standard stroke protocol parameters: 0.4 mm axial non-contrast scan pitch 0.55, 125 kVp, 300 mA, 1 s rotation time, caudocranial direction. Slice thickness for image reconstruction was 1 mm. Clot attenuation through the region of interest (ROI) within the clots was measured on axial and coronal reconstructions, recording the mean, maximum, and minimum Hounsfield Unit values.

Next, the phantoms with the clots were imaged using MRI within six hours of the CT scan. Each phantom was positioned inside a 20-channel standard head coil of a 1.5 T MRI-Scanner with the long axis of the Eppendorf tube in the AP direction, i.e., orthogonal to the magnetic field. The clot was then reimaged using a susceptibility-weighted imaging sequence with image acquisition in transverse orientation, i.e., parallel to the tube axis, covering a 3-dimensional block with an edge length of 90 × 199 × 250 mm (FH x AP x RL) (frequency encoding RL direction), reconstructed to voxels with an edge length of 1.1 × 0.32 × 0.32 mm [measured: 41 slices 2.2 mm thick (FH), pixel edge length 1.0 × 0.85 mm (AP x RL)]. Contrast parameters were: multi-echo 3D FFE sequences with T1 enhancement (i.e., gradient echo sequences with spoiling of the spin echo component), TR = 52 ms, 4 echoes with TEs of 12, 23, 34, 45 ms, a flip angle of 20°, modulus and phase image acquisition. For evaluation, the images were transferred to the PACS system and viewed on a clinical-grade monitor. Two reconstructed coronal slices cutting through the center of the clots were selected for further examination. A blooming artifact was defined as a signal drop that blurred and exceeded the clot boundaries, similar to its clinical appearance. Out of the 57 cases, there were two clots with doubtful positive susceptibility, which were included in the “BA yes” group because the periphery was affected.

### Statistical analysis

No a-priori sample size estimation was performed. In all, 57 clot analogs were acquired as described above. All statistical analyses were performed using SPSS version 26/2019 software (IBM). Continuous variables have been described in terms of the mean, standard deviation (SD), median, interquartile range (IQR), and range. We used the Mann-Whitney U test to test the differences in the continuous variables. Statistical significance was *P* ≤ .05. All reported P values were two-sided.

Decision trees were used in two parts of the results: to determine which HU-parameter (mean, maximum, minimum from axial or coronal reconstructions) was best associated with the iron content in clots (Figure S3), and to determine the iron threshold for the BA appearance (Fig. 2). In both cases, the settings of the CHAID decision-tree were: maximum depth: auto, minimum child size: 5, minimum parent size: 5, 10-fold cross-validation, alpha = 0.05. This analysis was Bonferroni adjusted. The sensibility and specificity for the iron cut-off associated with susceptibility signal was analyzed using an optimal Binning Minimum Description Length Principle (MDLP). Finally, receiver operating characteristic (ROC) curve analysis was used to assess predictors power for susceptibility signal in MRI for the total of clots (*n* = 57) and for the subgroup of clots with intermediate attenuation (*n* = 23). Area under ROC (AUC) was reported to indicate predictive power. Additionally, Spearman correlation (Spearman ρ) with 95% CI was performed to correlate CT attenuation (average axial) with iron and RBC. Logistic regression was performed to investigate the relationship between pH and clot composition.

## Electronic supplementary material

Below is the link to the electronic supplementary material.

Supplementary Material 1

## Data Availability

The data that support the findings of this study are available on request from the corresponding author AVG.

## Abbreviations

RBC: Red blood cells
BA: Blooming artifact
NCCT: Non-contrast CT
HU: Hounsfield Units
MRI: Magnetic resonance imaging
SWI: Susceptibility-weighted imaging
ms: Milliseconds
ROC: Receiver operating characteristic
