# Supplementary material for: Ironing out differences in attenuation and blooming artifact in acute stroke thrombi
Source: Sci Rep. 2025 Jan 2;15:103. doi: 10.1038/s41598-024-83916-0 (PMC11697157; doi:10.1038/s41598-024-83916-0)
Supplement: Supplementary file 1 — Supplementary Material 1 [file 41598_2024_83916_MOESM1_ESM.docx]

Ironing out differences in attenuation and blooming artifact in acute stroke thrombi

Original Article

Aglae Velasco Gonzalez (1), Dennis Görlich (2), Boris Buerke (1), Cristina Sauerland (2), Norbert Meier (5), Manfred Fobker (3), Ray McCarthy (6), Astrid Jeibmann (4), Walter Heindel (1), Andreas Faldum (2), Harald Kugel (5)

**Supplementary Material**

- - - **Figure S1** Boxplots illustrating the distribution of variables included in Table 1.
    - **Figure S2** Boxplots showing the distribution of variables analyzed in Table 2.
    - **Figure S3** Decision tree for iron content prediction based on the presence or absence of blooming artifact.
    - **Figure S4** Boxplots displaying the distribution of variables presented in Table 3, comparing two histological types of blooming artifact-negative thrombi (hypoattenuated and intermediate CT groups).
    - **Figure S5** Boxplots illustrating the subgroup analysis presented in Supplemental Table S1, highlighting the internal characteristics of the intermediate CT clot group and comparing BA-negative and BA-positive thrombi.
    - **Table S1** Subgroup analysis: internal characteristics of intermediate CT clot group (47.6–74.6 HU).

Supplementary Figure S1

Boxplots illustrating the distribution of variables included in Table 1. These variables include attenuation, RBC content, iron levels, water content, pH, sodium (Na), and partial oxygen pressure (pO₂).

Supplementary Figure S2

Boxplots showing the distribution of variables analyzed in Table 2. These include attenuation, RBC content, iron levels, and water content, comparing thrombi with and without blooming artifact (BA).

Supplementary Figure S3

**Figure S3** Decision tree for predicting the iron content from the presence or absence of the susceptibility signal (n = 57). The value of 1242 µg/g iron was chosen as the threshold value for determining whether the blooming artifact would appear for a clot analog. The RBC content allowed this decision tree to be further refined. Thus, thrombi with less than 1242 µg/g iron together with a histology of less than 90% RBC did not display the susceptibility signal (p = 0.003).

Supplementary Figure S4

Boxplots displaying the distribution of variables presented in Table 3, comparing two histological types of blooming artifact-negative thrombi (hypoattenuated and intermediate CT groups).

Supplementary Figure S5

Boxplots displaying the distribution of variables presented in Table 3, comparing two histological types of blooming artifact-negative thrombi (hypoattenuated and intermediate CT groups).

Supplementary Table S1

**Table S1** Subgroup analysis: internal characteristics of the intermediate CT clot group

|  | Intermediate attenuation thrombi  (47.6 – ≤ 74.6, n = 23) | | |
| --- | --- | --- | --- |
| Parameter | **BA-negative**  (n = 10) | **BA-positive**  (n = 13) | P-Value |
| Attenuation (HU)^1^  Median  IQR  Range | 62.6  55.9, 70  53.5 – 72.5 | 62.7  55.5, 71.7  51.8 – 74.6 | 0.9273 |
| RBC  Median  IQR  Range | 88  81.5, 93.8  68.3 – 99.5 | 75.6  64.2, 94.4  52.9 – 99.9 | 0.3128 |
| Iron (µg/g)  Median  IQR  Range | 1108  887, 1166  799 – 2290 | 1626  1324, 1675  906 – 2625 | 0.0255 |
| Water  Median  IQR  Range | 40.7  38.7, 71  25 – 73.8 | 72.7  69.5, 75.3  35.3 – 80.7 | 0.0255 |
| pH  Median  IQR  Range | 7.440  7.263, 7.580  7.124 – 7.6 | 6.824  6.677, 7.177  6.519 – 7.27 | < 0.001 |
| Na (mmol/L)  Median  IQR  Range | 17.9  16.8, 23  12.9 – 155 | 14.9  13.4, 151.4  10.7 – 154.3 | 0.5629 |
| pO2 (mmHg)  Median  IQR  Range | 199  195, 205  193 – 250 | 210  197, 243  191 – 254 | 0.2080 |

All parameter values are percentage values unless otherwise specified. ^1^Mean clot attenuation from 1 mm-axial non-contrast CT reconstructions. RBC: red blood cells; BA: blooming artifact. P-Value: Mann-Whitney U Test. Exact Sig (2-sided test) instead of asymptotic Sig (2-sided test).
